# Deep Machine Learning Might Aid in Combating Intensive Care Unit-Acquired Weakness

**DOI:** 10.7759/cureus.58963

**Published:** 2024-04-24

**Authors:** Chinmaya K Panda, Habib Md R Karim

**Affiliations:** 1 Anaesthesiology, Critical Care, and Pain Medicine, All India Institute of Medical Sciences, Raipur, Raipur, IND; 2 Anesthesiology, Critical Care, and Pain Medicine, All India Institute of Medical Sciences, Guwahati, Guwahati, IND

**Keywords:** weakness, intensive care unit, critical illness, artificial intelligence, machine learning

## Abstract

Secondary muscle weakness in critically ill patients like intensive care unit (ICU)-associated weakness is frequently noted in patients with prolonged mechanical ventilation and ICU stay. It can be a result of critical illness, myopathy, or neuropathy. Although ICU-acquired weakness (ICU-AW) has been known for a while, there is still no effective treatment for it. Therefore, prevention of ICU-AW becomes the utmost priority, and knowing the risk factors is crucial. Nevertheless, the pathophysiology and the attributing causes are complex for ICU-AW, and proper delineation and formulation of a preventive strategy from such vast, multifaceted data are challenging. Artificial intelligence has recently helped healthcare professionals understand and analyze such intricate data through deep machine learning. Hence, using such a strategy also helps in knowing the risk factors and their weight as contributors, applying them in formulating a preventive path for ICU-AW worth trials.

## Editorial

While advancements in contemporary medical practices have yielded notable reductions in intensive care unit (ICU) mortality rates, persistent challenges remain regarding post-ICU morbidity and the resultant impairment in quality of life, thus warranting further investigation and targeted interventions. The generalized muscle weakness arising during an ICU stay, with no discernible alternative etiology aside from the acute illness or its therapeutic interventions, is designated as "intensive care unit-acquired weakness" (ICU-AW) [1]. The entity bears importance owing to its impact on patient outcomes; it worsens acute morbidity and one-year mortality and increases healthcare-related costs [2]. It may even impact five-year morbidity and mortality [3].

The prevalence of ICU-AW varies widely among studies, with a systematic review reporting a median prevalence of 33%, highlighting predominant diaphragmatic involvement [4]. ICU-AW manifests as a generalized, symmetrical neuromuscular impairment primarily impacting proximal limb musculature, with concurrent involvement of respiratory musculature. Notably spared are facial and ocular muscle groups, suggesting a pattern consistent with critical illness polyneuropathy and myopathy. Muscle tone exhibited reduction, concomitant with observations of normal to reduced deep tendon reflexes [5]. ICU-AW is categorized into critical illness myopathy (CIM), critical illness polyneuropathy (CIP), and critical illness neuromyopathy (CINM).

The principal pathophysiological observation in the peripheral nerves of ICU-AW patients entails axonal degeneration without demyelination. Contributing factors include microvascular alterations within the endoneurium induced by sepsis, facilitating increased vascular permeability, and subsequent infiltration of neurotoxic agents into nerve terminals [6,7]. In CIM, early-onset muscle atrophy is a prominent feature. Various mechanisms, including inflammation, immobilization, endocrine stress responses, the rapid development of nutritional deficits, compromised microcirculation, and denervation, may collectively contribute to muscle protein degradation, particularly myosin, leading to muscle wasting [8,9]. Within muscular tissue, notable findings include significant myonecrosis, loss of myosin filaments, alteration in the actin: myosin ratio, disruption of muscle architecture, vacuolization, and phagocytic activity targeting myofilaments [9,10].

A systematic review evaluating the associated factors for ICU-AW noted that higher severity of illness as measured by Acute Physiology and Chronic Health Evaluation II score, multiple organ failure, systemic inflammatory response syndrome, sepsis, use of neuromuscular blocking agents, and aminoglycosides, electrolyte disturbances, hyperglycemia, high lactate level, parenteral nutrition and use of norepinephrine and duration of mechanical ventilation were significant contributor [11]. The meta-analysis also found female gender as a risk factor. Another meta-analysis showed a significant association between ICU-AW and corticosteroid use [12]. A logistic regression analysis showed that vasoactive medications, the duration of vasoactive medication use, and cumulative norepinephrine dose were independently associated with ICU-AW [13].

Multifactorial risk factors for ICU-AW are categorized into non-modifiable and modifiable factors. Non-modifiable risk factors include elevated severity illness scores, systemic inflammatory response syndrome, multiple organ failure, prolonged mechanical ventilation, extended critical illness duration, prolonged immobilization, elevated lactate levels, and advanced age. Modifiable risk factors encompass hyperglycemia, corticosteroid administration, neuromuscular blockade usage, vasoactive medications, vasopressors, aminoglycosides, vancomycin administration, and heightened levels of inflammatory mediators [14-17].

Artificial learning in healthcare is gaining popularity owing to the ability to apply deep machine learning strategies for complex healthcare data, and it can be applied in clinical practice to assist in diagnosis and treatment suggestions [18]. Wang et al. have identified significant risk factors for ICU-AW through iterative machine learning techniques and its prevention and treatment [19]. The authors categorized patients on the 14th day into ICU-AW and non-ICU-AW groups. Clinic-demographic, treatment, and support-related data, duration of mechanical ventilation, length of ICU stay, and rehabilitation therapy were considered variables, and their relationships with ICU-AW were examined. The authors developed a multilayer perceptron neural network model and assessed its predictive performance for ICU-AW. The study found that the model was effective in ICU-AW detection with an area under the curve of 0.941, sensitivity of 92.2%, and specificity of 82.7% [19]. The model found that higher ages, comorbidities, supports, and adverse events during ICU stay contributed to the development of ICU-AW; length of ICU stay and mechanical ventilation duration were the most critical factors. Interestingly, rehabilitation therapy was found to contribute to ICU-AW negatively, and sedation was found to impact it adversely [19].

Current guidelines advocate for the clinical diagnosis of ICU-AW through bedside assessment of muscle strength utilizing the Medical Research Council sum score [20]. This scoring system assigns a value ranging from 0 (indicating no contraction) to 5 (representing normal muscle strength) for each of the 12 muscle groups assessed. The cumulative score spans from 0 to 60, with ICU-AW being identified by a total score below 48. The scoring, however, does not depict the etiology and cannot differentiate between CIP and CIM. Alternative approaches, such as handheld dynamometry and handgrip strength assessment, have demonstrated favorable reproducibility in evaluating ICU-AW [21]. However, ultrasound findings have shown a limited correlation with ICU-AW diagnosis within the ICU setting [22]. Various methodologies exist for assessing respiratory muscle strength, including volitional and non-volitional techniques [23]. Although trans diaphragmatic pressure measurement following magnetic phrenic nerve stimulation offers valuable insights, its practical application for routine use is challenging [24].

Neurophysiological assessments encompass nerve conduction studies, which evaluate nerve conduction velocities, compound motor action potentials (CMAPs), sensory nerve action potentials (SNAPs), and electromyography (EMG). Diminished CMAPs and SNAPs alongside normal or near-normal nerve conduction velocities characterize CIP. CIM presents with brief duration, diminished amplitude motor unit potentials on EMG, and reduced CMAPs upon direct muscle stimulation. Four different electrophysiological clusters have been identified by Baum et al. for prognostication of ICU-AW [25]. Muscle biopsy is the gold standard for elucidating muscle involvement and distinguishing between ICU-AW and alternative diagnoses. Percutaneous muscle biopsy represents a readily feasible bedside procedure within the critical care milieu [26]. To date, biomarkers have not been validated; however, growth differentiation factor 15 (GDF-15), a new mediator involved in ICU-AW pathology, holds a promising future. Computed tomography, dual-energy X-ray absorptiometry, magnetic resonance imaging, and neutron activation analysis offer reliable means of detecting ICU-AW, albeit their implementation presents logistical challenges. Bioelectrical impedance measurements, which assess body composition, are susceptible to interference from factors such as edema, skin temperature variations, and patient positioning, limiting their interpretive accuracy within the critical care domain [27].

Short-term consequences encompass prolonged ICU admission, extended mechanical ventilation duration, failure in extubation, and elevated rates of ICU and hospital mortality. Various mechanisms, including respiratory muscle weakness, pharyngeal dysfunction, and symptomatic aspiration, contribute to augmented morbidity and mortality. Furthermore, healthcare-associated hospitalization expenses demonstrate a 30.5% increase, underscoring a causal association between ICU-AW and adverse clinical outcomes. The enduring ramifications of ICU-AW on physical function and health-related quality of life were initially investigated among acute respiratory distress syndrome survivors, revealing substantial declines in both parameters over five years. Predominant complaints among survivors included generalized fatigue and weakness, findings corroborated by additional research endeavors. Beyond muscle strength, pertinent factors potentially influencing outcomes encompass proprioception, gait balance, spatial attention, cognitive function, mental health, central nervous system dysfunction, pain, and entrapment neuropathy [2,28].

Like the causation, the preventive strategies for ICU-AW are multi-dimensional. The cornerstone of therapy for preventing ICU-AW lies in the aggressive management of sepsis, a leading cause of increased ICU stay and prolonged mechanical ventilation. While anti-inflammatory therapy has not demonstrated efficacy, targeting GDF-15 holds promise for mitigating muscle atrophy, thereby preventing ICU-AW. The optimal approach to blood glucose control remains a topic of debate; however, in critically ill patients, maintaining moderate hyperglycemia has been associated with a reduced incidence of ICU-AW compared to stringent glycemic control [29]. Early enteral nutrition is advocated over parenteral administration as a preventive measure against ICU-AW [16,30]. Needham et al. demonstrated that early hypocaloric enteral nutrition enhances physical function one-year post-ICU discharge [31]. Effective sedation promotes early mobilization in the ICU [32].

On the other hand, early mobilization facilitates optimal glycemic control and mitigates ICU-AW risk [33]. Findings from a systematic review and meta-analysis and exposure to corticosteroids for the dosage and duration should be limited [12]. Findings by Wang et al. indicate that minimizing the sedation and applying rehabilitation therapies and strategies to reduce the ICU stay and mechanical ventilation duration will likely provide a preventive action plan for ICU-AW [19]. Systematic review and meta-analysis indicate a possible role of early rehabilitation in preventing ICU-AW development [34]. While the role of early active mobilization alone for mitigating ICU-AW remains doubtful [35], combining early mobilization with early nutrition or neuromuscular stimulation appears promising [36,37].

However, a recent randomized controlled trial, i.e., TEAM study, involving mechanically ventilated adult patients, reported higher adverse events in the early mobilization group than usual care, necessitating further investigation into early mobilization strategies [35]. Interestingly, another randomized controlled trial evaluating the adjunctive use of neuromuscular electrical stimulation alongside early mobilization showed superior outcomes, including reduced ICU-AW incidence, enhanced muscle strength, and shorter hospital stay [37]. A systematic review assessing pharmacological interventions, including oxandrolone, growth hormone, propranolol, immunoglobulin, and glutamine therapy, initially considered promising for managing ICU-AW, failed to provide sufficient evidence to endorse their routine adoption in clinical practice [38]. Certain investigations have reported favorable impacts on functional exercise capacity following post-ICU physical rehabilitation, diverging from contrasting findings in other studies. Nonetheless, a systematic review failed to substantiate the benefits of post-ICU physical rehabilitation due to the limited quality of existing evidence and significant heterogeneity among studies [39].

Recently, healthcare and medicine practice has seen a data-driven paradigm shift. Efficient deep machine learning and artificial intelligence are helping devise algorithms to predict complex pathologies effectively and develop preventive strategies [40,41]. Application of such technological advancement might also help in anticipating and preparing preventive strategies for ICU-AW, as shown by Wang et al. [19]. Nevertheless, we will require multiple such studies enrolling different types of patients from various ethnicities. 

To conclude, ICU-AW has a multi-dimensional etiology and complex pathophysiology. In such a scenario, artificial intelligence might help us analyze complex data, predict at-risk patients, and formulate a preventive action plan. The study by Wang et al. shows us a path. Still, we will require multi-center collaboration to create big data and apply deep machine learning to validate the modality and find other hidden components, if any.
